# First-trimester aggregate index of systemic inflammation and adverse pregnancy outcomes in hospitalized women with threatened miscarriage: a single-center retrospective cohort study

**DOI:** 10.1186/s12884-026-09789-5

**Published:** 2026-08-22

**Authors:** Murat Haksever, Uğurcan Zorlu, Şengül Kara Eroğlu, Hakkı Şerbetçi, Şükrü Bakırcı, Esra Karataş, Dilek Şahin

**Affiliations:** 1 Department of Perinatology, Şanlıurfa Training and Research Hospital, Şanlıurfa, Türkiye; 2 Department of Obstetrics and Gynecology, Ministry of Health Ankara Bilkent City Hospital, University of Health Sciences, Ankara, Türkiye; 3 Perinatology Clinic, Ministry of Health Ankara Bilkent City Hospital, Ankara, Türkiye; 4 Department of Perinatology, Dr. İsmail Fehmi Cumalıoğlu City Hospital, Tekirdağ, Türkiye

**Keywords:** Threatened miscarriage, Aggregate index of systemic inflammation, AISI, Systemic immune-inflammation index, Miscarriage, Preterm birth, Prognostic model

## Abstract

**Background:**

Complete blood count-derived inflammatory indices have been associated with miscarriage, but the incremental value of the aggregate index of systemic inflammation (AISI) beyond established indices such as the systemic immune-inflammation index (SII) remains uncertain. We examined the association and prognostic performance of first-trimester AISI in hospitalized women with threatened miscarriage.

**Methods:**

This single-center retrospective cohort study included 312 women hospitalized with threatened miscarriage between January 2020 and December 2022. The primary outcome was a composite of miscarriage before 14 weeks, preterm birth before 37 weeks with or without preterm prelabor rupture of membranes (PPROM), or fetal demise from 14 weeks onward. AISI was analyzed continuously on the log2 scale. Multivariable logistic regression, restricted cubic splines, receiver operating characteristic analysis with DeLong comparisons, calibration, 500-resample bootstrap internal validation, and decision curve analysis were performed.

**Results:**

Adverse outcomes occurred in 134 women (42.9%). Median AISI was higher in the adverse-outcome group than in women with term birth (339.9 [IQR 250.2-432.1] vs. 236.6 [167.0-313.9]; *p* < 0.001). Each doubling of AISI was associated with higher adjusted odds of an adverse outcome (adjusted odds ratio 4.46, 95% CI 2.59–7.67; *p* < 0.001), with no evidence of nonlinearity (*p* = 0.499). AISI showed moderate discrimination (AUC 0.717, 95% CI 0.661–0.774), which was not significantly different from SII (AUC 0.696; DeLong *p* = 0.299). The exploratory AISI cut-off of 287.3 had 66.4% sensitivity (95% CI 58.1–73.9%) and 69.1% specificity (95% CI 62.0-75.4%). Adding AISI to the clinical plus C-reactive protein model increased the bootstrap-corrected AUC from 0.811 to 0.853 and reduced the corrected Brier score from 0.176 to 0.153; the incremental decision-curve benefit was modest.

**Conclusions:**

First-trimester AISI was independently associated with adverse pregnancy outcomes in this selected hospitalized cohort and added prognostic information to clinical variables and C-reactive protein. However, AISI alone had only moderate discrimination and was not superior to SII. These findings do not support a stand-alone clinical cut-off or a specific management intervention; prospective multicenter validation is required.

**Supplementary Information:**

The online version contains supplementary material available at https://doi.org/10.1186/s12884-026-09789-5.

## Background

Threatened miscarriage, commonly defined by vaginal bleeding with a closed cervical os during early pregnancy, affects a substantial proportion of pregnancies and is associated with increased risks of early pregnancy loss, preterm birth and other adverse outcomes [1–5]. The syndrome is etiologically heterogeneous, and early bleeding may coexist with embryonic or fetal, maternal, placental or cervical factors [2]. Consequently, a single biomarker is unlikely to capture all clinically relevant pathways.

Complete blood count-derived indices have been evaluated as inexpensive markers of systemic inflammatory burden. The neutrophil-to-lymphocyte ratio (NLR), systemic immune-inflammation index (SII) and systemic inflammation response index (SIRI) have shown variable associations with miscarriage and early pregnancy loss [6–9]. These indices are mathematically related and may also be affected by acute bleeding, physiological stress, obesity, smoking, occult infection and chronic disease.

AISI combines neutrophil, monocyte, platelet and lymphocyte counts and therefore incorporates additional cellular components compared with NLR or SII [10]. Recent studies have examined AISI in threatened miscarriage and in pregnancies complicated by PPROM [11, 12]. A recent cohort of women with threatened miscarriage reported an association between AISI and miscarriage, but did not establish superiority over established indices for later pregnancy complications [11]. Inflammatory dysregulation has also been proposed as a biologically plausible, but non-specific, contributor to miscarriage [13]. Thus, the novelty of AISI should be considered incremental rather than absolute.

The present study evaluated a larger cohort of hospitalized women with threatened miscarriage and a broader spectrum of pregnancy outcomes. The primary objectives were to determine whether admission AISI was associated with a composite adverse outcome after adjustment for established clinical factors and C-reactive protein (CRP), to compare AISI formally with other inflammatory indices, and to assess the incremental model performance, calibration and exploratory clinical net benefit of adding AISI.

## Methods

### Study design and setting

**Fig. 1:**
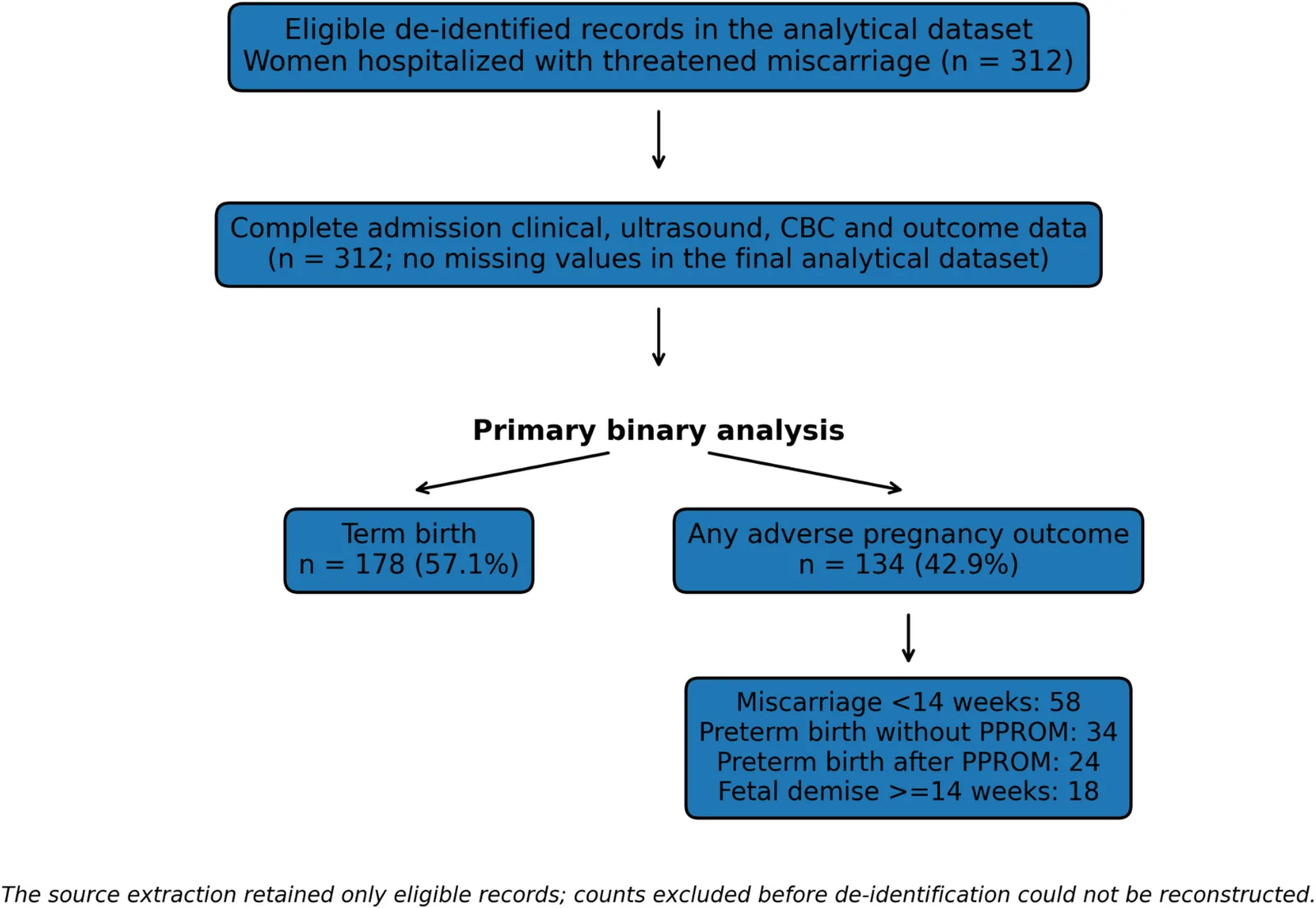
Analytical cohort and outcome ascertainment. The source extraction retained only eligible records; therefore, criterion-specific exclusion counts before de-identification could not be reconstructed

This single-center retrospective cohort study was conducted at the Perinatology Clinic of Ankara Bilkent City Hospital, a tertiary referral center. Electronic records of women hospitalized with threatened miscarriage between January 2020 and December 2022 were reviewed. The de-identified analytical file contained 312 women who met all eligibility criteria and had complete outcome data. The source extraction retained only eligible records; therefore, the number initially screened and the criterion-specific exclusion counts before de-identification could not be reconstructed (Fig. 1).

### Study population and viability assessment

Women with a singleton intrauterine pregnancy admitted at 6 + 0 to 13 + 6 weeks because of vaginal bleeding were eligible. Threatened miscarriage required a closed cervical os and pregnancy viability confirmed either at the admission ultrasound or at a short-interval follow-up examination. Fetal cardiac activity was not visualized on the initial scan in 38 women; all 38 had viability confirmed on follow-up before inclusion. The variable was therefore renamed “fetal cardiac activity not visualized initially” and was not treated as fetal death at enrollment.

Exclusion criteria were multiple gestation, a chromosomal anomaly documented during follow-up, active infection or systemic inflammatory disease documented in the admission record, autoimmune disease, malignancy, chronic hematologic disease, systemic corticosteroid or immunosuppressive treatment, missing admission laboratory data, or unavailable pregnancy outcome. Genetic testing was performed according to clinical indications rather than universally; consequently, the exclusion applied to documented chromosomal diagnoses and does not establish genetic normality in every pregnancy.

### Clinical variables

Maternal age, body mass index (BMI), gravidity, parity, smoking, comorbidity, previous miscarriage, previous preterm birth, gestational age at blood sampling, subchorionic hematoma, cervical length, assisted reproductive technology/in vitro fertilization conception, progesterone treatment and hospital stay were abstracted. Bleeding severity was extracted from the treating clinician’s categorical documentation as mild or moderate-to-severe. A validated quantitative bleeding score, pad count or measured blood loss was not available. Cervical length was analyzed continuously; no categorical short-cervix threshold was used.

Active infection or inflammatory disease was defined by a clinician-documented diagnosis in the admission record, supported when available by clinical, laboratory or microbiological findings. Universal microbiological, TORCH or serological testing was not performed. Serial complete blood counts, thrombophilia testing, anticoagulant treatment, endocrine testing and placental histopathology were not recorded systematically in the analytical dataset.

### Laboratory measurements and inflammatory indices

Peripheral venous blood obtained at hospitalization was analyzed using the institution’s standardized automated hematology workflow. White blood cell, neutrophil, lymphocyte, monocyte and platelet counts, hemoglobin, red cell distribution width and CRP were recorded. All indices were recalculated from the same admission sample: NLR=neutrophils/lymphocytes; platelet-to-lymphocyte ratio (PLR)=platelets/lymphocytes; monocyte-to-lymphocyte ratio (MLR)=monocytes/lymphocytes; SII=platelets x neutrophils/lymphocytes; SIRI=neutrophils x monocytes/lymphocytes; and AISI=neutrophils x monocytes x platelets/lymphocytes. Recalculation reproduced the patient-level values exactly.

### Outcome definitions

The primary binary outcome was any adverse pregnancy outcome. Outcome categories were defined as: term birth at 37 + 0 weeks or later; miscarriage before 14 + 0 weeks; preterm birth from 24 + 0 to 36 + 6 weeks without PPROM; preterm birth from 24 + 0 to 36 + 6 weeks after PPROM; and fetal demise from 14 + 0 weeks onward. The term “intrauterine exitus” was replaced by “fetal demise.” The specific indications for preterm birth without PPROM were not retained reliably in the analytical extraction and were therefore not analyzed. The composite outcome was used to provide adequate event numbers for multivariable analysis, while category-specific results were retained descriptively because the conditions are pathophysiologically heterogeneous.

### Statistical analysis

Continuous variables were summarized as mean +/- standard deviation or median (interquartile range), and categorical variables as n (%). Across the five outcome categories, one-way analysis of variance or Kruskal-Wallis tests were used for continuous variables and chi-square tests for categorical variables. The final analytical dataset had no missing values.

The primary association analysis used multivariable binary logistic regression with term birth versus any adverse outcome. AISI and CRP were log2-transformed; the AISI odds ratio therefore represents a doubling rather than an arbitrary 100-unit increment. Covariates were specified a priori on clinical grounds and were not selected by stepwise testing: maternal age, BMI, gestational age at sampling, moderate-to-severe bleeding, fetal cardiac activity not visualized initially, subchorionic hematoma, cervical length, previous miscarriage, previous preterm birth, progesterone treatment and CRP. A sensitivity model additionally included smoking, comorbidity and ART/IVF. The linearity of log2(AISI) was assessed with a restricted cubic spline. Variance inflation factors (VIFs) were calculated for model covariates. Because the inflammatory indices share mathematical components, they were not entered simultaneously; their Spearman correlations are reported in Supplementary Table S1.

ROC AUCs and 95% confidence intervals were estimated for AISI and comparator indices. Correlated AUCs were compared using the DeLong method [14]. Exploratory Youden cut-offs were reported with bootstrap 95% confidence intervals; sensitivity and specificity confidence intervals were calculated using the Wilson method. Cut-offs were considered data-derived and not clinical decision thresholds.

Two prognostic models were compared: a baseline model containing the prespecified clinical variables and CRP, and the same model plus AISI. Discrimination, Brier score and calibration were evaluated. Optimism-corrected performance was estimated using 500 bootstrap resamples. Repeated stratified 10-fold out-of-fold probabilities were used to display calibration and decision curves. Decision curve analysis was restricted to threshold probabilities of 10%-40% and interpreted as exploratory [15]. The reporting of model development and internal validation was structured with reference to TRIPOD recommendations [16]. No single threshold was designated for clinical use.

No a priori sample-size calculation was performed because the study used a fixed retrospective cohort. The primary model contained 12 parameters and 134 adverse events, corresponding to approximately 11.2 events per parameter. This ratio is reported descriptively and was not treated as proof of sample-size adequacy, in accordance with contemporary prediction-model guidance [17]. The original multinomial model was removed because the smallest outcome category contained only 18 events and could not support comprehensive adjustment without substantial overfitting. Analyses were performed using Python 3.13 with SciPy, statsmodels and scikit-learn. Two-sided *p* < 0.05 was considered statistically significant.

### Ethics approval

The Ankara Bilkent City Hospital Clinical Research Ethics Committee No. 1 approved the study (TABED 1-24-583; September 25, 2024) and waived written informed consent because the study used anonymized retrospective data. The study was conducted in accordance with the Declaration of Helsinki.

## Results

All 312 women in the analytical dataset had complete outcome follow-up (Fig. 1). Baseline maternal and obstetric characteristics are presented in Table 1. Moderate-to-severe bleeding, fetal cardiac activity not visualized initially, subchorionic hematoma, shorter cervical length and previous preterm birth differed across outcome categories.

**Table 1:** Baseline maternal and obstetric characteristics according to pregnancy outcome

Variable	Total (*n* = 312)	Term birth (*n* = 178)	Miscarriage (*n* = 58)	Preterm without PPROM (*n* = 34)	Preterm after PPROM (*n* = 24)	Fetal demise (*n* = 18)	*p* value
Maternal age, years	29.9 ± 5.7	29.2 ± 5.4	30.9 ± 6.1	30.1 ± 5.8	31.4 ± 6.0	31.7 ± 6.5	0.095
BMI, kg/m²	26.8 ± 3.8	26.3 ± 3.6	27.4 ± 4.1	27.1 ± 4.0	27.8 ± 4.3	27.6 ± 3.8	0.130
Gravidity	2 (2–3)	2 (2–3)	2 (2–3)	3 (2–4)	2 (2–3)	2 (1–2)	0.001
Parity	1 (0–1)	1 (0–1)	0 (0–1)	1 (0–1)	1 (0–2)	0 (0–1)	0.312
Nulliparity	148 (47.4)	79 (44.4)	32 (55.2)	15 (44.1)	11 (45.8)	11 (61.1)	0.465
Smoking	52 (16.7)	25 (14.0)	12 (20.7)	7 (20.6)	5 (20.8)	3 (16.7)	0.693
Comorbidity	38 (12.2)	17 (9.6)	10 (17.2)	5 (14.7)	4 (16.7)	2 (11.1)	0.523
Prior miscarriage	96 (30.8)	46 (25.8)	26 (44.8)	12 (35.3)	8 (33.3)	4 (22.2)	0.077
Prior preterm birth	28 (9.0)	10 (5.6)	3 (5.2)	7 (20.6)	5 (20.8)	3 (16.7)	0.006
Gestational age at blood sampling, weeks	8.4 ± 1.6	8.6 ± 1.5	7.9 ± 1.6	8.3 ± 1.6	8.1 ± 1.6	8.0 ± 1.7	0.026
Moderate-to-severe bleeding	102 (32.7)	44 (24.7)	34 (58.6)	12 (35.3)	8 (33.3)	4 (22.2)	< 0.001
Fetal cardiac activity not visualized initially	38 (12.2)	8 (4.5)	20 (34.5)	3 (8.8)	3 (12.5)	4 (22.2)	< 0.001
Subchorionic hematoma	118 (37.8)	54 (30.3)	32 (55.2)	15 (44.1)	11 (45.8)	6 (33.3)	0.011
Cervical length, mm	34.8 ± 4.6	35.7 ± 4.1	35.4 ± 4.2	31.8 ± 4.8	30.9 ± 5.0	34.2 ± 4.5	< 0.001
ART/IVF pregnancy	42 (13.5)	22 (12.4)	9 (15.5)	6 (17.6)	3 (12.5)	2 (11.1)	0.908
Progesterone treatment	226 (72.4)	126 (70.8)	44 (75.9)	27 (79.4)	18 (75.0)	11 (61.1)	0.618
Hospital stay, days	2 (1–3)	2 (1–3)	3 (2–4)	2 (1–3)	2 (1–2)	2 (1–2)	< 0.001

*PPROM* Preterm prelabor rupture of membranes, *ART* Assisted reproductive technology, *IVF* in vitro fertilization

Values are mean +/- standard deviation, median (interquartile range), or n (%). Overall p values were obtained using one-way ANOVA, Kruskal-Wallis or chi-square tests, as appropriate

Admission complete blood count variables, inflammatory markers and calculated indices are presented in Table 2. Median AISI was 236.6 (IQR 167.0-313.9) in women with term birth and 339.9 (250.2-432.1) in women with an adverse outcome (*p* < 0.001). SII, SIRI and NLR were also higher in the adverse-outcome group. All women whose fetal cardiac activity was not visualized initially had subsequent ultrasound confirmation of viability before inclusion.

**Table 2:** Admission complete blood count, inflammatory markers and calculated indices according to pregnancy outcome

Variable	Total (*n* = 312)	Term birth (*n* = 178)	Miscarriage (*n* = 58)	Preterm without PPROM (*n* = 34)	Preterm after PPROM (*n* = 24)	Fetal demise (*n* = 18)	*p* value
WBC, 10⁹/L	8.0 ± 1.0	7.8 ± 1.0	8.4 ± 1.0	8.1 ± 1.1	8.2 ± 0.9	8.0 ± 1.0	< 0.001
Neutrophils, 10⁹/L	5.00 ± 1.12	4.76 ± 1.09	5.42 ± 1.05	5.26 ± 1.13	5.33 ± 1.04	5.15 ± 1.12	< 0.001
Lymphocytes, 10⁹/L	2.15 ± 0.47	2.22 ± 0.48	2.10 ± 0.44	2.03 ± 0.48	2.09 ± 0.40	1.97 ± 0.53	0.046
Monocytes, 10⁹/L	0.47 ± 0.12	0.45 ± 0.12	0.50 ± 0.13	0.48 ± 0.12	0.49 ± 0.13	0.49 ± 0.12	0.053
Platelets, 10⁹/L	262.6 ± 46.4	256.8 ± 46.0	278.4 ± 53.6	259.1 ± 34.3	280.9 ± 37.6	250.8 ± 41.6	0.005
Hemoglobin, g/dL	12.16 ± 0.99	12.25 ± 0.98	11.96 ± 0.96	12.17 ± 0.99	12.01 ± 0.94	12.11 ± 1.16	0.347
RDW, %	13.19 ± 0.97	13.17 ± 1.03	13.26 ± 0.83	13.25 ± 0.94	13.25 ± 1.00	13.00 ± 0.87	0.865
CRP, mg/L	1.71 (0.83–3.56)	1.46 (0.69–3.08)	2.25 (1.21–4.18)	1.75 (0.75–4.07)	2.38 (1.02–5.50)	2.08 (1.18–3.68)	0.007
NLR	2.33 (1.84–2.95)	2.14 (1.68–2.65)	2.71 (2.11–3.24)	2.63 (1.93–3.54)	2.34 (2.04–3.13)	2.64 (2.05–3.15)	< 0.001
PLR	122.0 (100.5–152.0)	115.4 (94.3–142.3)	134.9 (110.8–159.9)	130.2 (111.2–158.6)	142.7 (116.5–153.2)	125.6 (108.8–150.7)	0.004
MLR	0.22 (0.17–0.27)	0.21 (0.15–0.25)	0.24 (0.20–0.29)	0.24 (0.18–0.30)	0.22 (0.19–0.27)	0.23 (0.21–0.33)	0.004
SII	612.7 (454.6–776.0)	539.3 (425.1–687.2)	728.6 (598.4–874.0)	658.4 (539.6–897.5)	655.5 (612.2–845.1)	648.8 (536.9–775.7)	< 0.001
SIRI	1.08 (0.78–1.41)	0.94 (0.69–1.24)	1.27 (0.97–1.57)	1.24 (0.93–1.70)	1.23 (0.98–1.49)	1.31 (1.06–1.52)	< 0.001
AISI	274.1 (206.2–376.8)	236.6 (167.0–313.9)	347.0 (258.4–440.5)	336.5 (236.9–422.0)	347.5 (273.0–426.3)	314.9 (248.0–345.1)	< 0.001

Values are mean +/- standard deviation or median (interquartile range)

*AISI* Aggregate index of systemic inflammation, *CRP* C-reactive protein, *MLR* Monocyte-to-lymphocyte ratio, *NLR* Neutrophil-to-lymphocyte ratio, *PLR* Platelet-to-lymphocyte ratio, *SII* Systemic immune-inflammation index, *SIRI* Systemic inflammation response index

Pregnancy outcome distribution and gestational age at outcome are presented in Table 3. Term birth occurred in 178 women (57.1%), miscarriage before 14 weeks in 58 (18.6%), preterm birth without PPROM in 34 (10.9%), preterm birth after PPROM in 24 (7.7%) and fetal demise from 14 weeks onward in 18 (5.8%).

**Table 3:** Pregnancy outcome distribution and gestational age at outcome

Outcome definition	*n*	%	Gestational age, weeks, median (IQR)
Term birth, >=37 + 0 weeks	178	57.1	38.9 (37.8–40.1)
Miscarriage, < 14 + 0 weeks	58	18.6	9.9 (8.1–11.6)
Preterm birth without PPROM, 24 + 0–36 + 6 weeks	34	10.9	32.2 (27.6–34.0)
Preterm birth after PPROM, 24 + 0–36 + 6 weeks	24	7.7	30.1 (26.9–33.3)
Fetal demise, >=14 + 0 weeks	18	5.8	23.9 (20.5–30.9)

In the prespecified multivariable model, each doubling of AISI was associated with higher odds of an adverse outcome (aOR 4.46, 95% CI 2.59–7.67; *p* < 0.001) after adjustment for clinical variables and CRP (Table 4). The association remained similar after additional adjustment for smoking, comorbidity and ART/IVF (aOR 4.54, 95% CI 2.61–7.91; *p* < 0.001; Supplementary Table S2). The restricted cubic spline showed no evidence that a nonlinear function improved model fit (p for nonlinearity = 0.499; Supplementary Figure S1). Model VIFs were low (maximum 1.10).

**Table 4:** Multivariable logistic regression for any adverse pregnancy outcome

Predictor	Adjusted odds ratio (95% CI)	*p* value
Maternal age, per year	1.09 (1.04–1.16)	0.001
BMI, per kg/m²	1.07 (0.99–1.16)	0.103
Gestational age at sampling, per week	0.70 (0.57–0.85)	< 0.001
Moderate-to-severe bleeding	2.48 (1.29–4.76)	0.006
FCA not visualized initially	5.56 (1.97–15.70)	0.001
Subchorionic hematoma	4.43 (2.28–8.60)	< 0.001
Cervical length, per 5-mm increase	0.43 (0.30–0.62)	< 0.001
Prior miscarriage	2.41 (1.24–4.66)	0.009
Prior preterm birth	9.06 (2.85–28.80)	< 0.001
Progesterone treatment	0.99 (0.50–1.96)	0.968
CRP, per doubling	1.62 (1.26–2.10)	< 0.001
AISI, per doubling	4.46 (2.59–7.67)	< 0.001

The primary model included all variables shown. AISI and CRP were log2-transformed. The AISI odds ratio therefore represents a doubling. No stepwise variable selection was used. The outcome combined miscarriage, preterm birth with or without PPROM and fetal demise

**Fig. 2:**
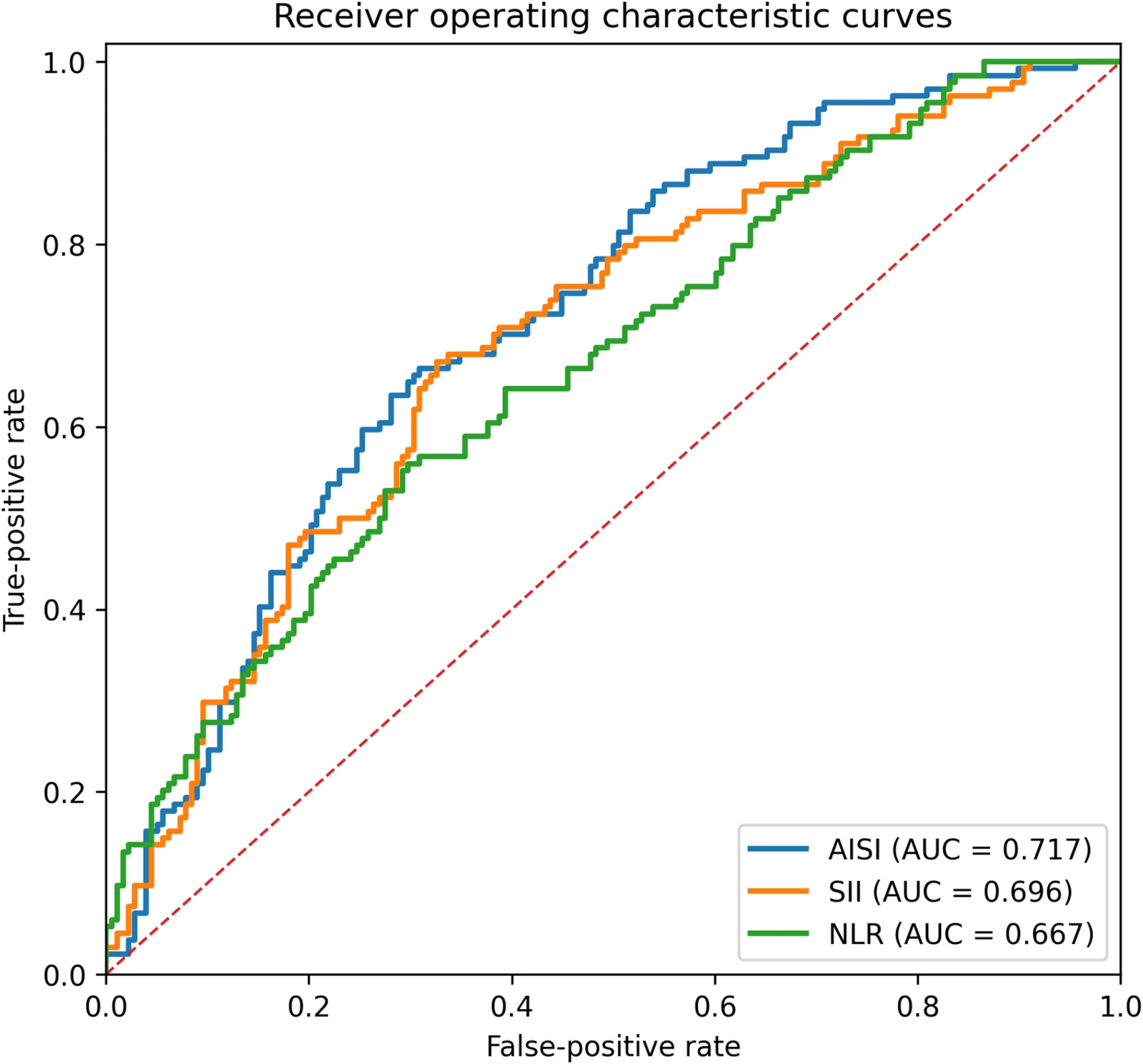
Receiver operating characteristic curves for AISI, SII and NLR. AISI showed moderate discrimination; the AISI-SII AUC difference was not statistically significant (DeLong *p* = 0.299)

AISI had an AUC of 0.717 (95% CI 0.661–0.774), compared with 0.696 (0.638–0.755) for SII and 0.667 (0.607–0.727) for NLR (Table 5; Fig. 2). The AISI-SII difference was not significant (DeLong *p* = 0.299), whereas AISI exceeded NLR (*p* = 0.041). The exploratory AISI cut-off was 287.3 (bootstrap 95% CI 219.1–332.0), with sensitivity 66.4% (95% CI 58.1–73.9%) and specificity 69.1% (95% CI 62.0-75.4%). The wide cut-off interval confirms that it should not be considered a validated clinical threshold.

**Table 5:** ROC performance of inflammatory indices for any adverse pregnancy outcome

Index	AUC (95% CI)	Exploratory cut-off (bootstrap 95% CI)	Sensitivity, % (95% CI)	Specificity, % (95% CI)	DeLong *p* vs. AISI
AISI	0.717 (0.661–0.774)	287.3 (219.1–332.0)	66.4 (58.1–73.9)	69.1 (62.0-75.4)	Reference
SII	0.696 (0.638–0.755)	620.6 (536.4-727.1)	67.2 (58.8–74.5)	67.4 (60.2–73.9)	0.299
SIRI	0.696 (0.638–0.754)	1.16 (0.84–1.23)	61.9 (53.5–69.7)	70.8 (63.7–77.0)	0.109
NLR	0.667 (0.607–0.727)	2.53 (1.85–3.05)	56.0 (47.5–64.1)	70.2 (63.1–76.5)	0.041
PLR	0.626 (0.564–0.689)	125.6 (105.6-135.3)	60.4 (52.0-68.3)	62.9 (55.6–69.7)	< 0.001
MLR	0.626 (0.565–0.688)	0.27 (0.15–0.27)	38.1 (30.3–46.5)	80.9 (74.5–86.0)	< 0.001
CRP	0.615 (0.552–0.677)	0.65 (0.65–5.98)	92.5 (86.8–95.9)	23.0 (17.5–29.7)	0.018

AUC confidence intervals were calculated using the DeLong method. Cut-off confidence intervals were obtained by bootstrap resampling; sensitivity and specificity confidence intervals used the Wilson method. All cut-offs are exploratory and are not recommended clinical thresholds

**Fig. 3:**
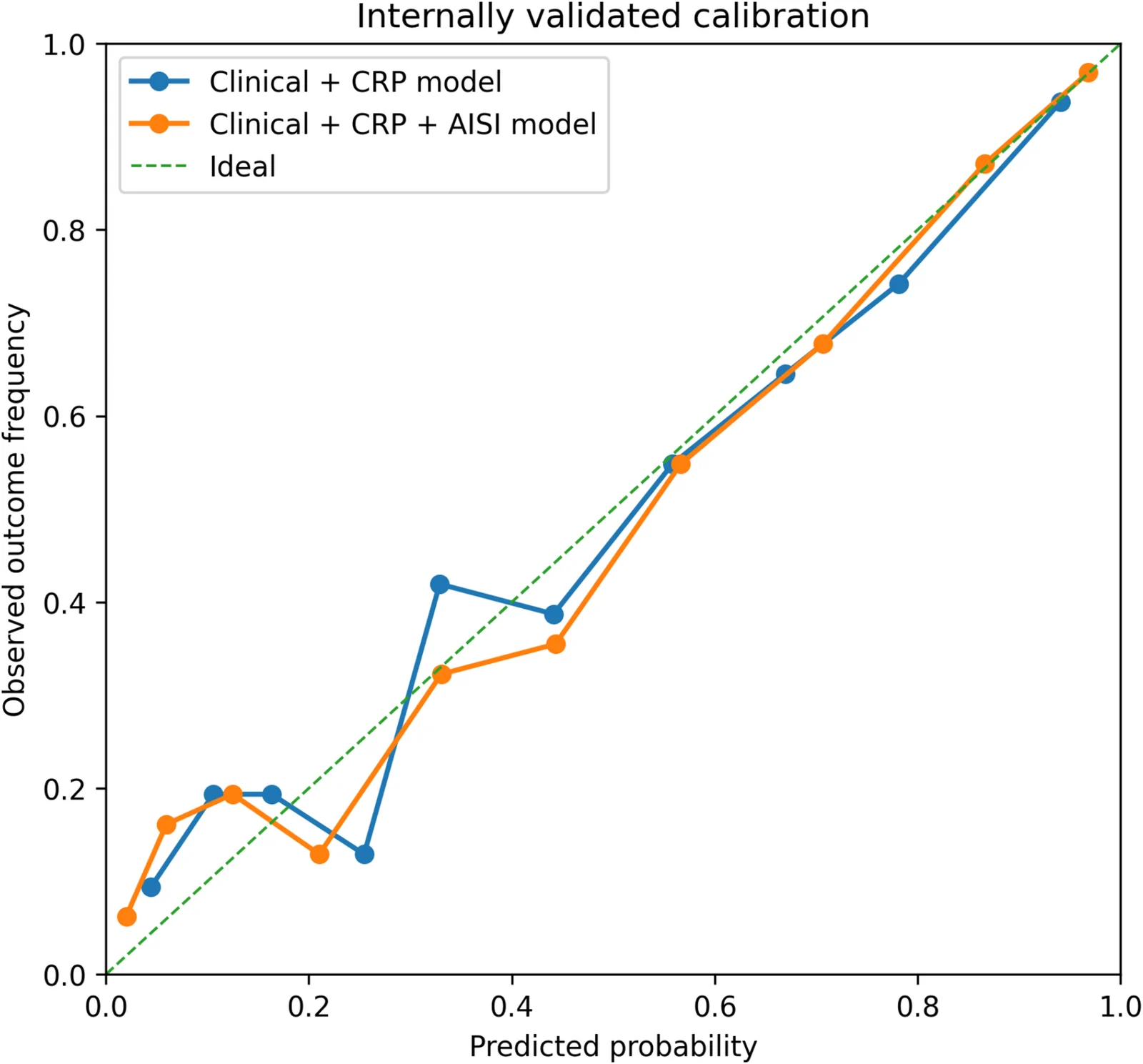
Calibration curves based on repeated stratified 10-fold out-of-fold probabilities. Both models showed some overfitting, reflected by calibration slopes below 1 after internal validation

The apparent AUC increased from 0.835 for the clinical plus CRP model to 0.876 after adding AISI (DeLong *p* = 0.004), and the Brier score decreased from 0.161 to 0.137. After 500-resample bootstrap correction, the corresponding AUCs were 0.811 and 0.853 and Brier scores were 0.176 and 0.153 (Table 6). Optimism-corrected calibration slopes were 0.872 and 0.864, respectively. The cross-validated calibration curves are shown in Fig. 3.

**Table 6:** Performance and internal validation of the clinical model before and after adding AISI

Model	Apparent AUC	Bootstrap-corrected AUC	Apparent Brier score	Bootstrap-corrected Brier score	Bootstrap-corrected calibration slope
Clinical variables + CRP	0.835	0.811	0.161	0.176	0.872
Clinical variables + CRP + AISI	0.876	0.853	0.137	0.153	0.864

Internal validation used 500 bootstrap resamples. Lower Brier scores indicate lower overall prediction error. The apparent AUC difference was significant by DeLong testing (*p* = 0.004), but externally validated performance is unknown

**Fig. 4:**
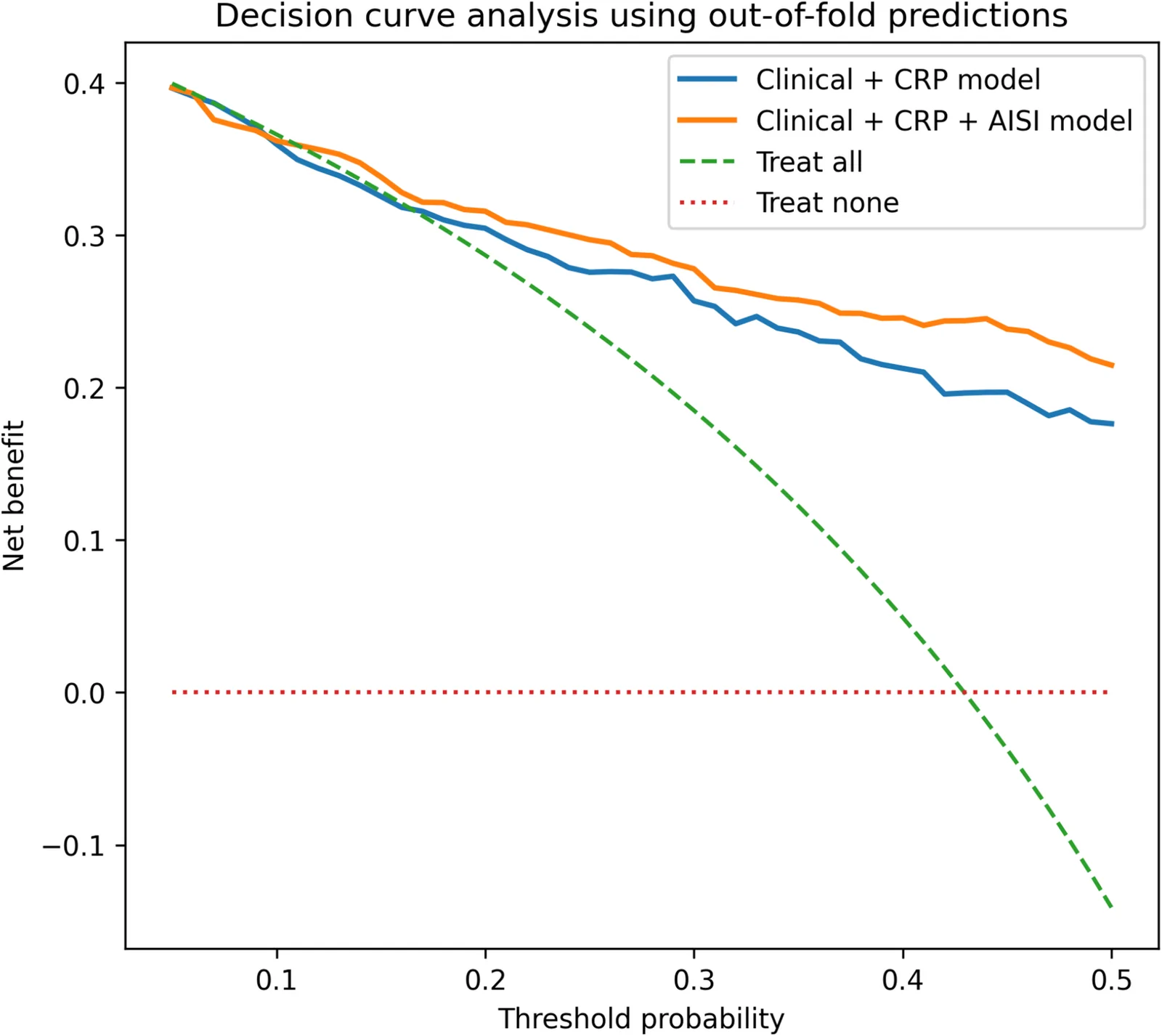
Decision curve analysis based on out-of-fold probabilities. The incremental net benefit of adding AISI was small at lower thresholds and modest at thresholds above approximately 20%

Decision curve analysis based on out-of-fold probabilities showed little difference at thresholds of 10%-15%, and modestly higher net benefit for the AISI model at thresholds above approximately 20% (Fig. 4). This exploratory result does not demonstrate that AISI-guided intervention improves outcomes.

## Discussion

In this single-center cohort of hospitalized women with threatened miscarriage, higher admission AISI was independently associated with a composite adverse pregnancy outcome. AISI showed moderate univariable discrimination and added prognostic information to a model containing clinical variables and CRP. However, its AUC was not significantly different from SII, the data-derived cut-off was unstable, and the improvement in decision-curve net benefit was modest. These qualifications materially limit claims of superiority or immediate clinical usefulness.

Previous studies have associated SII and related indices with miscarriage. Turgut et al. reported an SII cut-off of 883.95 with approximately 63% sensitivity and specificity among women with threatened miscarriage [6]. Soysal et al. also reported higher SII in threatened miscarriage and an AUC of 0.727 for predicting miscarriage within that cohort [7]. Çallıoğlu et al. found associations involving SIRI and SII, whereas Gong et al. reported limited predictive value for several systemic inflammatory indices [8, 9]. Differences in gestational age, outcome definition, acute bleeding, laboratory platforms and control selection may explain part of this heterogeneity.

A recent study specifically examining AISI in threatened miscarriage reported a cut-off of 414.63, 72.7% sensitivity and 57.3% specificity for miscarriage, but admission AISI did not differentiate complicated from uncomplicated ongoing pregnancies [11]. Our lower exploratory cut-off of 287.3 and broader composite outcome are not directly interchangeable with that study. More importantly, the formal AISI-SII comparison in our cohort was nonsignificant. Therefore, the present contribution is the broader outcome follow-up, direct comparator testing, comprehensive adjustment and internal validation, rather than demonstration of a clinically important superiority of AISI over SII.

AISI incorporates monocytes and platelets in addition to neutrophils and lymphocytes [10]. This may provide incremental information when these cellular compartments vary independently, but the very high correlations with SIRI and SII confirm that the indices largely reflect overlapping information. AISI should therefore be viewed as a composite summary of routine blood counts, not a distinct mechanistic biomarker. Acute hemorrhage, stress-mediated leukocyte redistribution, occult infection and underlying maternal conditions may influence the component counts and create associations that are not specific to pregnancy pathophysiology.

The adjusted association with AISI does not establish that systemic inflammation caused miscarriage, PPROM, preterm birth or fetal demise. Although inflammatory dysregulation is biologically plausible in miscarriage [13], placental function, trophoblast invasion, fetal growth, preeclampsia, Doppler findings and placental histopathology were not measured systematically. Mechanistic interpretations involving placental dysfunction therefore remain hypotheses rather than findings of this study.

The clinical implications must also remain limited. No evidence-based intervention was tested for patients with elevated AISI, and there is no demonstrated management action that would improve outcome. The previous 15% threshold, which produced very low specificity, was removed. At most, AISI could be investigated as a risk-enrichment variable within a broader assessment that includes bleeding characteristics, ultrasound findings, cervical length and obstetric history. It should not be used to trigger surveillance, treatment or patient counseling as a stand-alone measure.

The interval between first-trimester sampling and later outcomes is an additional limitation. Only the admission blood count was available systematically; serial inflammatory indices and measurements at PPROM or delivery were not analyzed. The observed association therefore concerns baseline prognostic information, not a persistent inflammatory phenotype. Likewise, universal microbiological testing, placental pathology, thrombophilia evaluation, anticoagulant exposure and endocrine assessment were unavailable.

The composite outcome improved statistical efficiency but combined clinically distinct events. Early miscarriage, spontaneous or indicated preterm birth, PPROM and later fetal demise have different causal structures. The specific indications for non-PPROM preterm birth were not available, and category-specific adjusted analyses were not sufficiently supported by the smallest event groups. The composite result should therefore be interpreted as a general adverse-outcome association rather than a common causal mechanism.

Strengths include complete outcome data in the final cohort, recalculation of all inflammatory indices from patient-level blood counts, formal comparison of correlated AUCs, clinically broader covariate adjustment, assessment of functional form and collinearity, and internal validation with calibration and decision-curve evaluation.

Limitations include retrospective selection and information bias, inability to reconstruct the number initially screened or excluded before de-identification, restriction to a hospitalized tertiary-center population, clinician-recorded rather than quantitatively standardized bleeding severity, incomplete etiologic characterization, a single inflammatory measurement, residual confounding, use of a heterogeneous composite outcome and absence of external validation. The model performance remains susceptible to optimism despite internal validation, and no clinical-effect study was conducted.

## Conclusions

In this single-center retrospective cohort of hospitalized women with threatened miscarriage, first-trimester AISI was associated with adverse pregnancy outcomes after adjustment for clinical factors and CRP. AISI provided moderate discrimination comparable to SII and modest incremental model information, but it did not establish causality, a validated clinical cut-off or an effective management strategy. Prospective multicenter studies with standardized etiologic assessment, serial measurements and external validation are required before AISI can be considered for routine risk stratification.

## Supplementary Information

Supplementary Material 1.

## Data Availability

The datasets generated and/or analyzed during the current study are not publicly available due to institutional and patient privacy restrictions but are available from the corresponding author on reasonable request.

## Abbreviations

AISI: aggregate index of systemic inflammation
aOR: adjusted odds ratio
ART: assisted reproductive technology
AUC: area under the receiver operating characteristic curve
BMI: body mass index
CI: confidence interval
CRP: C-reactive protein
FCA: fetal cardiac activity
IVF: in vitro fertilization
MLR: monocyte-to-lymphocyte ratio
NLR: neutrophil-to-lymphocyte ratio
PLR: platelet-to-lymphocyte ratio
PPROM: preterm prelabor rupture of membranes
RDW: red cell distribution width
ROC: receiver operating characteristic
SII: systemic immune-inflammation index
SIRI: systemic inflammation response index
VIF: variance inflation factor

## References

[1] Karimi A, Sayehmiri K, Vaismoradi M, Dianatinasab M, Daliri S. Vaginal bleeding in pregnancy and adverse clinical outcomes: a systematic review and meta-analysis. J Obstet Gynaecol. 2024;44(1):2288224. 10.1080/01443615.2023.2288224. 38305047

[2] Sotiriadis A, Papatheodorou S, Makrydimas G. Threatened miscarriage: evaluation and management. BMJ. 2004;329(7458):152–5. 10.1136/bmj.329.7458.152. PMC478228 15258071

[3] Ahmed SR, El-Sammani MEK, Al-Sheeha MA, Aitallah AS, Khan FJ, Ahmed SR. Pregnancy outcome in women with threatened miscarriage: a year study. Mater Sociomed. 2012;24(1):26–8. 10.5455/msm.2012.24.26-28. PMC3643802 23678307

[4] Dadkhah F, Kashanian M, Eliasi G. A comparison between the pregnancy outcome in women both with or without threatened abortion. Early Hum Dev. 2010;86(3):193–6. 10.1016/j.earlhumdev.2010.02.005. 20231080

[5] Hasan R, Baird DD, Herring AH, Olshan AF, Funk MLJ, Hartmann KE. Association between first-trimester vaginal bleeding and miscarriage. Obstet Gynecol. 2009;114(4):860–7. 10.1097/AOG.0b013e3181b79796. PMC2828396 19888046

[6] Turgut E, Yildirim M, Sakcak B, Ayhan SG, Tekin OM, Sahin D. Predicting miscarriage using systemic immune-inflammation index. J Obstet Gynaecol Res. 2022;48(3):587–92. 10.1111/jog.15156. 35040233

[7] Soysal C, Sarı H, Işıkalan MM, Özkaya EB, Ulaş Ö, Taşçı Y, Keskin N. Role of the systemic immune-inflammation index in threatened abortion patients and predicting of abortion. J Obstet Gynaecol Res. 2023;49(7):1723–8. 10.1111/jog.15655. 37088799

[8] Çallıoğlu N, Gül DK, Arslan İÖ, Geyikoğlu İ, Demirçivi E. Inflammatory markers in systemic immune-inflammatory index and inflammatory response index to predict early pregnancy loss. Saudi Med J. 2024;45(8):808–13. 10.15537/smj.2024.45.8.20240404. PMC11288500 39074885

[9] Gong H, Fang H, Jiang Q, Hua P, Shen Y, Chen Q. Evaluating the predictive value of systemic inflammation indices in early pregnancy loss: a retrospective observational study. BMC Immunol. 2025;26(1):100. 10.1186/s12865-025-00782-9. PMC12752213 41275118

[10] Zinellu A, Collu C, Nasser M, et al. The aggregate index of systemic inflammation (AISI): a novel prognostic biomarker in idiopathic pulmonary fibrosis. J Clin Med. 2021;10(18):4134. 10.3390/jcm10184134. PMC8466198 34575245

[11] Baştemur AG, Haliloğlu E, Tanaçan A, et al. Relationship between aggregate index of systemic inflammation with miscarriage and pregnancy complications. J Reprod Immunol. 2026;174:104869. 10.1016/j.jri.2026.104869. 41793938

[12] Özden Tokalıoğlu E, Tanaçan A, Öztürk Ağaoğlu M, et al. Aggregate index of systemic inflammation: a novel systemic inflammatory index for prediction of neonatal outcomes and chorioamnionitis in women with preterm premature rupture of membranes. Int J Gynaecol Obstet. 2025;168(2):640–9. 10.1002/ijgo.15868. 39157934

[13] Christiansen OB, Nielsen HS, Kolte AM. Inflammation and miscarriage. Semin Fetal Neonatal Med. 2006;11(5):302–8. 10.1016/j.siny.2006.03.001. 16682265

[14] DeLong ER, DeLong DM, Clarke-Pearson DL. Comparing the areas under two or more correlated receiver operating characteristic curves: a nonparametric approach. Biometrics. 1988;44(3):837–45. 10.2307/2531595. 3203132

[15] Vickers AJ, Elkin EB. Decision curve analysis: a novel method for evaluating prediction models. Med Decis Mak. 2006;26(6):565–74. 10.1177/0272989X06295361. PMC2577036 17099194

[16] Collins GS, Reitsma JB, Altman DG, Moons KGM. Transparent Reporting of a multivariable prediction model for Individual Prognosis or Diagnosis (TRIPOD): the TRIPOD statement. Diabet Med. 2015;32(2):146–54. 10.1111/dme.12654. 25600898

[17] Riley RD, Snell KIE, Ensor J, et al. Minimum sample size for developing a multivariable prediction model: Part II-binary and time-to-event outcomes. Stat Med. 2019;38(7):1276–96. 10.1002/sim.7992. PMC6519266 30357870

