# Supplementary material for: First-trimester aggregate index of systemic inflammation and adverse pregnancy outcomes in hospitalized women with threatened miscarriage: a single-center retrospective cohort study
Source: BMC Pregnancy Childbirth. 2026 Aug 22;26:1213. doi: 10.1186/s12884-026-09789-5 (PMC13692858; doi:10.1186/s12884-026-09789-5)
Supplement: Supplementary file 1 [file 12884_2026_9789_MOESM1_ESM.docx]

**Supplementary material**

**First-trimester aggregate index of systemic inflammation and adverse pregnancy outcomes in hospitalized women with threatened miscarriage: a single-center retrospective cohort study**

**Supplementary Table S1. Spearman correlations among complete blood count-derived inflammatory indices**

| **Index** | **AISI** | **SII** | **SIRI** | **NLR** | **PLR** | **MLR** |
| --- | --- | --- | --- | --- | --- | --- |
| **AISI** | 1.000 | 0.798 | 0.913 | 0.693 | 0.638 | 0.755 |
| **SII** | 0.798 | 1.000 | 0.670 | 0.873 | 0.772 | 0.381 |
| **SIRI** | 0.913 | 0.670 | 1.000 | 0.762 | 0.438 | 0.822 |
| **NLR** | 0.693 | 0.873 | 0.762 | 1.000 | 0.544 | 0.427 |
| **PLR** | 0.638 | 0.772 | 0.438 | 0.544 | 1.000 | 0.499 |
| **MLR** | 0.755 | 0.381 | 0.822 | 0.427 | 0.499 | 1.000 |

*All values are Spearman rank coefficients. The high correlations reflect shared mathematical components; the indices were not entered together in multivariable models.*

**Supplementary Table S2. Expanded sensitivity analysis for AISI**

| **Analysis** | **Adjusted OR for AISI doubling (95% CI)** | **p value** |
| --- | --- | --- |
| Primary model plus smoking, comorbidity and ART/IVF | 4.54 (2.61-7.91) | <0.001 |

*The expanded model also included maternal age, BMI, gestational age at sampling, bleeding severity, fetal cardiac activity not visualized initially, subchorionic hematoma, cervical length, previous miscarriage, previous preterm birth, progesterone treatment and log2(CRP).*

**Supplementary Figure S1. Restricted cubic spline for AISI**

*Adjusted odds ratios are shown relative to the median AISI. Shaded bands represent 95% confidence intervals. There was no evidence of nonlinearity (p=0.499).*
